# Identification of Risk Factors for Locoregional Recurrence in Breast Cancer Patients with Nodal Stage N0 and N1: Who Could Benefit from Post-Mastectomy Radiotherapy?

**DOI:** 10.1371/journal.pone.0145463

**Published:** 2015-12-21

**Authors:** Eunjin Jwa, Kyung Hwan Shin, Hyeon Woo Lim, So-Youn Jung, Seeyoun Lee, Han-Sung Kang, EunSook Lee, Young Hee Park

**Affiliations:** 1 Center for Breast Cancer, Research Institute and Hospital, National Cancer Center, Goyang, Korea; 2 Department of Radiation Oncology, Soonchunhyang University College of Medicine, Cheonan, Korea; 3 Departments of Radiation Oncology, Seoul National University College of Medicine, Seoul, Korea; 4 Department of Radiation Oncology, Soonchunhyang University College of Medicine, Seoul, Korea; INRS, CANADA

## Abstract

**Introduction:**

The locoregional recurrence (LRR) rate was reported as high as approximately 20% in stage I-II breast cancer following mastectomy. To investigate the risk factors for LRR in pT1–2N0-1 breast cancer patients treated with mastectomy but not radiation, and to define a subgroup of patients at high risk of LRR who may benefit from postmastectomy radiotherapy (PMRT).

**Methods and Materials:**

In total, 390 patients with pT1-2N0M0 (n = 307) and pT1-2N1M0 (n = 83) breast cancer who underwent total mastectomy without adjuvant radiotherapy from 2002 to 2011 were enrolled in the study.

**Results:**

After a median follow-up period of 5.6 years (range, 0.6–11.3 years), 21 patients had 18 systemic relapses and 12 LRRs including six in the chest wall and eight in the regional nodal area. The 5-year LRR-free survival (LRRFS) rates were 97.0% in pN0, 98.8% in pN1, and 97.4% in all patients. Multivariate analysis revealed that age < 50 years (Hazard Ratio, 11.4; *p* = 0.01) and no adjuvant chemotherapy (Hazard Ratio, 10.2; *p* = 0.04) were independent risk factors for LRR in pN0 patients. Using these factors, the 5-year LRRFS rates were 100% without any risk factors, 96.4% with one risk factor, and 86.7% with two risk factors. In pN1 patients, multivariate analysis revealed that having a hormone receptor negative tumor (Hazard Ratio, 18.3; *p* = 0.03) was the only independent risk factor for LRR. The 5-year LRRFS rates were 100.0% for luminal type, and 92.3% for non-luminal type cancer.

**Conclusion:**

Patients with pT1-2N0-1 breast cancer who underwent total mastectomy without PMRT could be stratified by nodal stage and risk factors for LRR. PMRT may have of value for node negative patients aged less than 50 years and who are not treated with adjuvant chemotherapy, and for non-luminal type patients with one to three positive nodes.

## Introduction

Several guidelines have recommended postmastectomy radiotherapy (PMRT) in breast cancer patients with tumors > 5 cm, four or more involved axillary lymph nodes (LNs), and invasion of the pectoral muscle or the surgical margins [1–3]. Adjuvant PMRT has been shown to reduce the rate of locoregional recurrence (LRR), and to improve overall survival (OS) rates in high-risk patients [4,5].

However, the usefulness of PMRT in early-stage breast cancer patients with tumors <5cm in size and three or fewer involved nodes (pT1–2N0-1) has not been clearly defined. The Early Breast Cancer Trialists Collaborative Group (EBCTCG) meta-analysis demonstrated no differences in the 10-year recurrence risk (21.1% (no RT) and 22.4% (RT)) in node negative patients who were treated with mastectomy, or in the 20-year breast cancer mortality rate after RT [6]. The Danish Breast Cancer Cooperative Group (DBCG) published a study on the value of PMRT in patients with one to three positive axillary LNs; the results showed that the 15-year LRR rate declined by 23% in patients after postoperative radiotherapy (RT), and the 15-year OS improved in 9% of patients [4,5]. However, these trials have been criticized for their limitations, including the use of chemotherapy, limited axillary dissection, and the use of old radiation techniques.

PMRT is not routinely recommended in breast cancer patients with stage of pT1–2N0-1, but, according to the National Comprehensive Cancer Network guidelines, may be considered for patients with multiple high-risk recurrence factors [1]. A number of adverse prognostic factors for LRR in early breast cancer patients have been reported [7–18], but the results are inconsistent, and specific adverse prognostic factors for LRR remain unclearly defined. Therefore, investigating LRR risk factors is important to identify early-stage breast cancer patients who might benefit from PMRT after mastectomy.

The purpose of this study was to identify the risk factors for LRR in pT1–2N0-1 breast cancer patients treated with mastectomy but not radiation, and to define a subgroup of patients at high risk for LRR who might benefit from PMRT.

## Materials and Methods

### Patient population

This study was a retrospective review of data in a breast cancer database that was established at the National Cancer Center, Korea. This study and the database were approved by the institutional review board of the National Cancer Center. Written informed consent was not obtained but patient information was anonymized and de-identified prior to analysis. In total, 390 breast cancer patients, pathologic stage T1-2N0-1M0, were enrolled. All patients had undergone total mastectomy with sentinel biopsy and/or axillary lymph node dissection without adjuvant RT between 2002 and 2011. The patients had been pathologically staged according to the sixth edition of the American Joint Committee on Cancer staging guidelines. Patient follow-up data, including age, menopausal status, pathological tumor size, and the number of LNs dissected, and pathological features including histological type, histological grade, estrogen receptor (ER), progesterone receptor (PR), and HER2 and Ki-67 status were obtained from electronic medical records. An immunohistochemistry assay was used to evaluate the expression of ER, PR, HER2, and Ki-67 markers. Tumors were defined based on the Allred score as ER- or PR-positive when at least 3/8 of the tumor cells examined had strong nuclear staining [19]. ER and PR status were categorized as hormone receptor (HR) positive when ER or PR staining was positive, and as HR negative when ER and PR staining were negative. Immunostaining for HER2 was considered positive in the case of strong (3+) membranous staining in at least 10% of tumor cells, or in the case of unequivocal amplification by fluorescence *in situ* hybridization (2+) [20]. To evaluate Ki-67 expression, the areas with the highest Ki-67 staining were investigated, and 15% was used as the cut-off value for Ki-67 to dichotomize the patients [21–23]. Patients were classified into immunohistochemistry-based molecular subgroups based on immunohistochemical features as follows: luminal A (HR+/HER2-/Ki-67<15%), luminal B1 (HR+/HER2-/Ki-67≥15%), luminal B2 (HR+/HER2+), HER2 (HR-/HER2+), and triple negative (HR-/HER2-). These subtypes were also grouped into luminal type (A, B1, B2) and non-luminal type (HER2 enriched, triple negative) tumors for further analysis.

### Statistical Analysis

LRR was defined as recurrent disease in the ipsilateral chest wall, or in the axillary, supraclavicular, infraclavicular, or internal mammary LNs. Local recurrence (LR) was defined as recurrent disease in the ipsilateral chest wall. Distant metastasis (DM) was defined as any recurrence except LRR. All LRRs were considered to be an event, regardless of whether they were the first site of failure or whether they were distant (DM). Patients who did not experience LRR were censored at the last follow-up or at the time of death. The actual LRR rates were calculated from the date of mastectomy using the Kaplan–Meier method, and differences between groups were compared using a two-sided log-rank test. Logistic regression was used to evaluate the association between covariates and the probability of LRR. Multivariate analyses were performed using the Cox proportional hazards model. All calculations were performed using SPSS version 18.0 (SPSS, Chicago, IL, USA). Two-tailed *P*-values ≤ 0.05 were considered statistically significant.

## Results

### Patient characteristics

Patient characteristics are summarized in Table 1. The median age at diagnosis of the 390 patients was 59 years (range, 37–87 years). All patients underwent total mastectomy and sentinel lymph node biopsy or axillary lymphadenectomy with clear resection margins. A median number of six LNs (range, 1–28) were removed in all patients, including four (range, 1–28) in patients with pN0, and 12 (range, 3–25) in patients with pN1. Fifty-five patients had one involved LN, 16 had two involved LNs, and 12 patients had three involved LNs. Adjuvant chemotherapy was delivered to 212 patients (54.4%) according to the physician’s discretion. Anthracycline based chemotherapy was predominantly used (n = 130) with additional taxane in selected cases (n = 16) in node negative patients. Twenty-five patients were treated with anthracycline-based regimen, and 41 patients received anthracycline combined taxane in pN1 stage. Adjuvant hormonal therapy was offered to 288 patients (pN0, n = 223; pN1, n = 65) with ER- or PR- positive tumors. Following surgery, trastuzumab was administered for one year to 19 patients with HER2 positive tumors, including ten (3.3%) with luminal B2 and nine (10.3%) with HER2 subtypes. Six patients with pN1 and 13 patients with no positive nodes received trastuzumab.

**Table 1:** Baseline characteristics.

		pN0 (n = 307)		pN1 (n = 83)		All (n = 390)		
		n	%	n	%	n		%
Age	<50 years	54	17.6	11	13.3	65		16.7
	≥50 years	253	82.4	72	86.7	325		83.3
	median (range)	59 years (37–87)		63 years (40–87)		59 years (37–87)		
Pathology	Invasive ductal	273	88.9	76	91.6	349	89.5	
	others	34	11.1	7	8.4	41	10.5	
Menopause	Premenopause	118	38.4	32	38.6	150	38.5	
	Postmenopause	189	61.6	51	61.4	240	61.5	
Pathologic T stage	1	195	63.5	33	39.8	228	58.5	
	2	112	36.5	50	60.2	162	41.5	
Histologic grade	Well-moderate	157	51.1	48	57.8	205	52.6	
	Poor	110	35.8	32	38.6	142	36.4	
	Unknown	40	13.0	3	3.6	43	11.0	
Lymphovascular invasion	No	183	59.6	50	60.2	233	59.7	
	Yes	124	40.4	33	39.8	157	40.3	
Molecular subtype	Luminal A	78	25.4	18	21.7	96	24.6	
	Luminal B1	32	10.4	16	19.3	48	12.3	
	Luminal B2	124	40.4	35	42.2	159	40.8	
	HER2	43	14.0	11	13.3	54	13.8	
	Triple negative	30	9.8	3	3.6	33	8.5	
Adjuvant chemotherapy	Yes	146	47.6	66	79.5	212	54.4	
	No	161	52.4	17	20.5	178	45.6	

### Relapse Patterns

**Fig 1:**
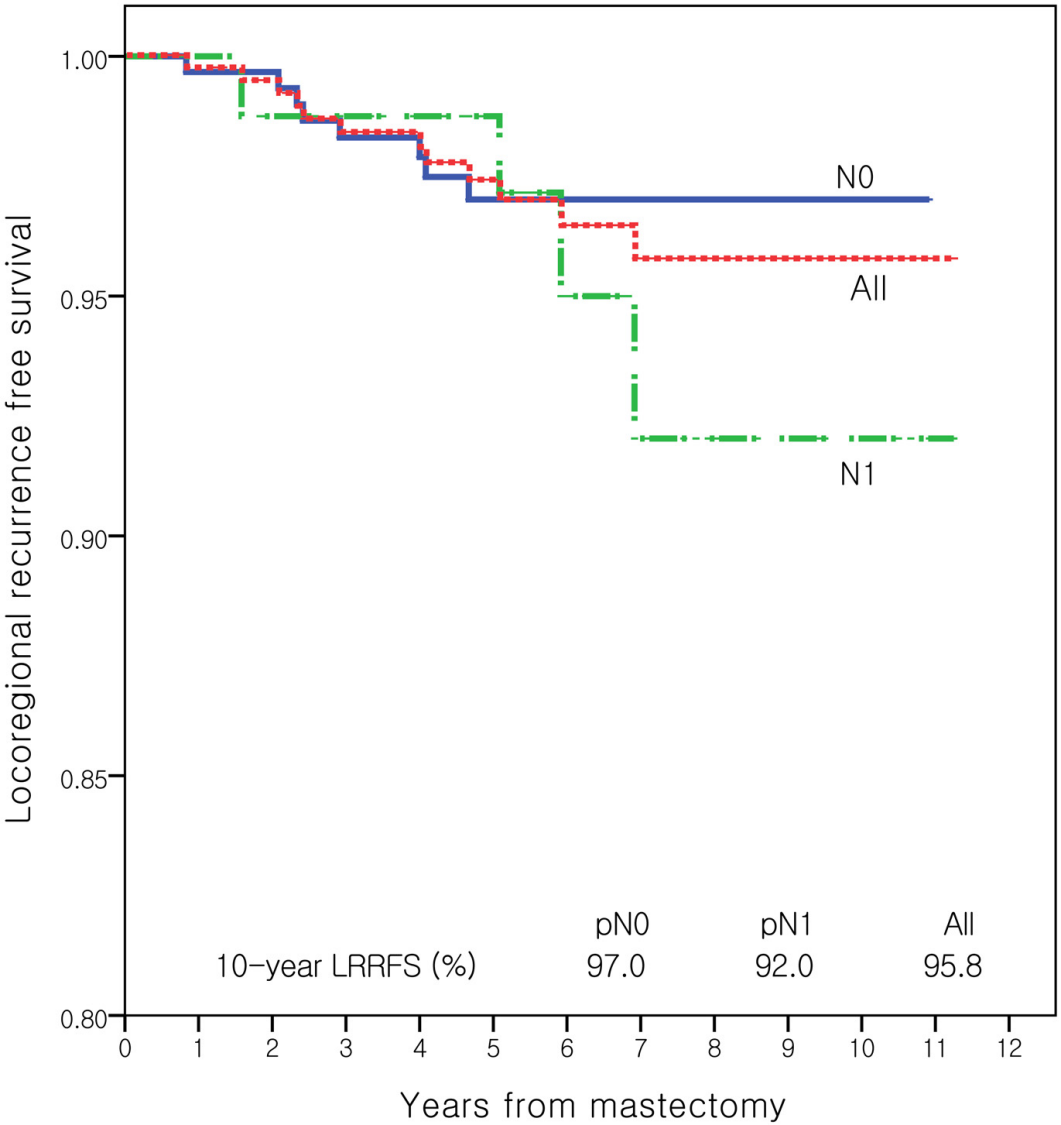
Kaplan-Meier curves for locoregional recurrence free survival (LRRFS) according to the pathologic nodal stage.

The median follow-up period was 5.6 years (range, 0.6–11.3 years). There were six chest wall recurrences, eight regional recurrences, 18 DMs, and 24 deaths that occurred during the follow-up period. Table 2 shows the recurrence site patterns. While LR rates were similar between patients with pN0 and pN1, patients with 1–3 positive nodes had more recurrences in the supraclavicular area and distant sites. The cumulative incidence of 5-year LR-free survival was 98.6%, LRR-free survival (LRRFS) was 97.8%, DM-free survival was 96.7%, and disease-free survival was 95.5%. LRRFS according to nodal stage is shown in Fig 1. The 5-year LRR-free survival (LRRFS) rates were 97.0% in pN0, 98.8% in pN1, and 97.4% in all patients.

**Table 2:** Recurrence sites as a factor of failures in stage N0, N1 and all patients.

	pN0 stage (n = 307)		pN1 stage (n = 83)		all (n = 390)	
	n	%	n	%	n	%
Local (Ipsilateral chest wall)	5	1.6	1	1.2	6	1.5
Regional	5	1.6	3	3.6	8	2.1
Axilla	3	1.0	1	1.2	4	1.0
Internal mammary	0	0.0	1	1.2	1	0.3
Supraclavicular	2	0.7	2	2.4	4	1.0
Locoregional	8	2.6	4	4.8	12	3.1
Distant	10	3.3	8	9.6	18	4.6

### Prognostic factors & risk group for LRR

**Fig 2:**
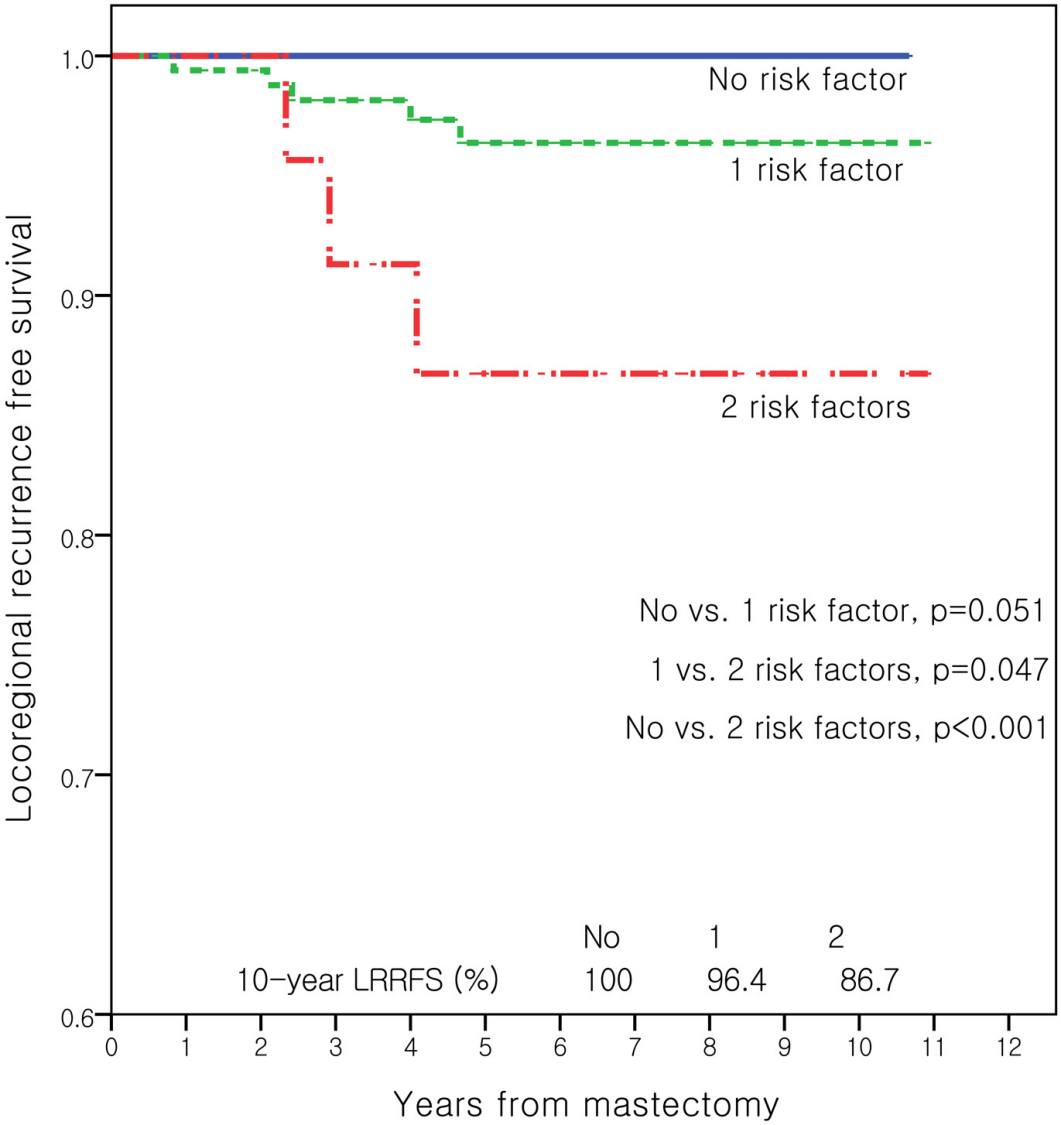
Kaplan-Meier curves for locoregional recurrence free survival (LRRFS) according to the number of risk factors in pN0 patients. Risk factors are age under 50 years and no use of adjuvant chemotherapy.

Prognosis analyses were performed separately according to pathologic nodal stage. Age, menopausal status, histologic grade, use of adjuvant chemotherapy, luminal type, pathologic T stage, lymphovascular invasion, and number of removed LNs were used for univariate analysis in node negative patients. No use of adjuvant chemotherapy (*p* = 0.04) and age under 50 years (*p* = 0.03) were the only significant risk factors for LRR in pN0 patients. Kaplan–Meier curves also showed that these two factors were significantly associated with LRRFS. The 5-year LRRFS rates were 99.3% in patients treated with adjuvant chemotherapy, and 94.8% in those without chemotherapy (*p* = 0.037). In terms of age, the 5-year LRRFS rates are 98.1% in patients aged 50 years or over and 91.9% in those aged 49 and under (*p* = 0.018). However, pathologic T stage (*p* = 0.91), luminal type (*p* = 0.35), and histologic grade (*p* = 0.39) were not significant prognostic factors for LRR in pN0 patients. Multivariate analyses showed the same results (Table 3). These analyses defined three risk groups as follows: low risk (no risk factor), intermediate risk (one risk factor), and high risk (two risk factors). Fig 2 shows that high-risk group had a significantly higher LRR than both the low-risk (*p* < 0.001) and intermediate-risk (*p* = 0.047) groups.

**Table 3:** Multivariate analysis for locoregional recurrence in stage N0, N1 patients.

Variable	pN0 stage (n = 307)		pN1 stage (n = 83)	
	Hazard ratio (95% CI)	p-value	Hazard ratio (95% CI)	p-value
Age (<50 vs. ≥50 years)	11.4 (2.4–55.4)	0.01	1.3 (0.1–12.8)	0.99
Adjuvant chemotherapy (not done vs. done)	10.2 (1.2–88.5)	0.04	1.1 (0.1–23.0)	0.97
Molecular subtype (non-luminal vs. luminal)	1.2 (0.2–7.3)	0.82	18.3 (1.3–257.9)	0.03
No. of positive nodes (2–3 vs. 0–1)	-	-	6.4 (0.6–73.2)	0.14

**Fig 3:**
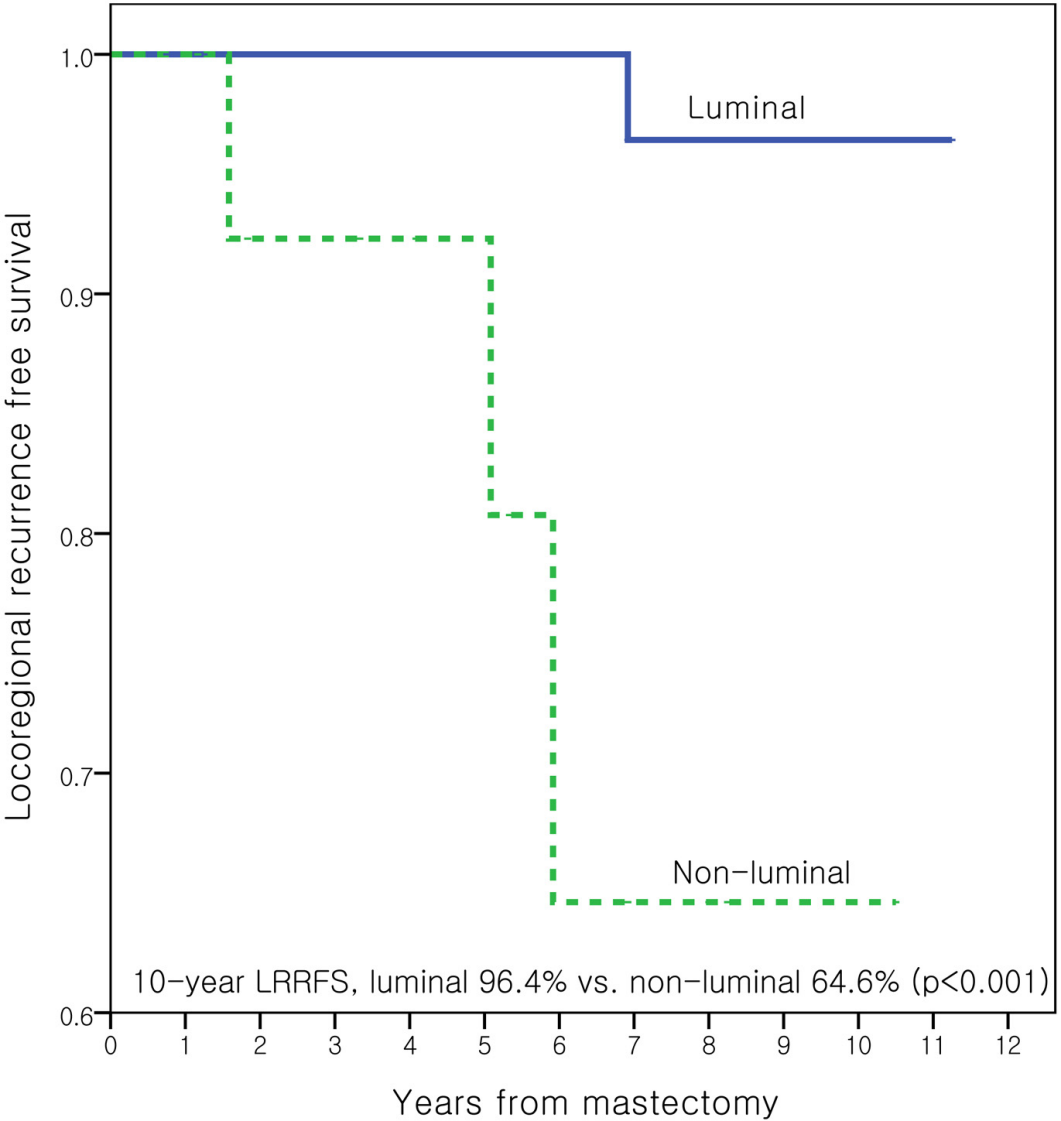
Kaplan-Meier curves for locoregional recurrence free survival (LRRFS) according to the luminal subtype in pN1 patients.

In pN1 patients, age, menopausal status, histologic grade, use of adjuvant chemotherapy, luminal type, pathologic T stage, lymphovascular invasion, number of LNs removed, and the number of LNs involved (1 vs. 2–3) were used for univariate analysis. Non-luminal type was the only significant prognostic factor for LRR (RR, 20.01; 95% CI 2.07–193.83; *p* = 0.01). Age (< 50 years vs. ≥ 50 years; *p* = 0.65), use of adjuvant chemotherapy (*p* = 0.80), pathologic T stage (*p* = 0.54), number of involved LNs (*p* = 0.10), and histologic grade (*p* = 0.49) were not significant prognostic factors for LRR in pN1 patients. Table 3 details the multivariate analysis for LRR in pN1 patients. Non-luminal subtype was the only independent prognostic factor for LRR in pN1 patients (hazard ratio, 18.3; 95% CI 1.3–257.9; *p* = 0.03). Fig 3 shows that the LRRFS was significantly higher in patients with luminal compared to non-luminal subtypes (*p* < 0.001). The 5-year LRRFS rates were 100.0% in patients with positive ER or PR and 92.3% in those without positive HR (*p* = 0.037).

## Discussion

Whether or not patients treated with mastectomy that have T1-2 disease and zero to three involved nodes should receive adjuvant systemic therapy remains controversial. Which patients might benefit from PMRT is also unknown. In this study, age under 50 years at diagnosis and no use of adjuvant chemotherapy were significant risk factors for LRR in patients with T1-2, node negative breast cancer treated with mastectomy but not radiation. The 10-year LRRFS rate was 86.7% in young node negative patients who were not treated with adjuvant chemotherapy. Having an HR negative tumor (10-year LRR rate, 64.6% vs. 96.4% in HR positive) was the only significant risk factor for LRR in pN1 patients treated with the same local treatment.

In addition to LN stage, previous studies have reported that young age, premenopausal status, molecular receptor status, lymphovascular invasion, margin status, use of systemic therapy, tumor size, and histologic grade are risk factors for LRR in postmastectomy patients with zero to three positive axillary LNs without adjuvant RT [7–18]. In the case of node negative breast cancer, several studies have shown that young age is a risk factor for LRR. In a subset analysis of 753 patients with T1-2 node-negative breast cancer, Sharma et al. reported that age <40 years was the only significant independent predictor of LRR [7], and the 10-year rates of LRR were 1.0% in patients >40 years, but 10.5% in patients 40 years or younger (*p* < 0.001). Abi-Raad et al. analyzed 1136 patients with T1-2N0 breast cancer, and again found that younger age (<50 years) was associated with increased risk for LRR (Hazard Ratio, 0.5; *p* = 0.018) [8]. They also reported that tumor size >2 cm, positive margins, young age, no systemic therapy, and lymphovascular invasion were poor prognostic factors for LRR in pN0 patients, with a 20% risk of LRR without PMRT in patients with three or more prognostic factors. However, other studies have found no association between age and LRR in node negative breast cancer patients [9–13].

Several investigators have reported different results in terms of systemic chemotherapy as a risk factor for LRR. Truong et al. found that no systemic therapy was associated with increased risk of LRR compared to systemic therapy (14.1% chemotherapy alone, 29.9% hormone therapy alone, 6.7% both) in patients with pT1-2N0 cancer (Hazard Ratio, 1.87; *p* = 0.01) [9]. The authors conducted recursive partitioning analysis to evaluate risk when multiple variables were combined, and they found that no systemic therapy was associated with a 10-year LRR of 23.2% compared with 9.2% for systemic therapy (*p* = 0.001) in patients with Grade 3 pT2 disease with no lymphovascular invasion. Wallgren et al. analyzed 1275 patients with node-negative disease in IBCSG trials randomized to receive either no adjuvant therapy (33%) or a single cycle of cyclophosphamide/methotrexate/ fluorouracil (66%), and found that the LRR risk increased significantly without adjuvant chemotherapy [16]. Among premenopausal patients, those who received chemotherapy had a 10-year cumulative incidence for LRR of 12% compared with 18% for no chemotherapy (*p* = 0.035). Among postmenopausal patients, they found no difference between those who received adjuvant chemotherapy compared to those who did not (10-year LRR: 10% vs. 14%; *p* = 0.18).

Triple negative and HER2 enriched subtypes are known risk factors for disease recurrence and survival. However, in patients with early-stage breast cancer (pT1–2N0-1), the impact of molecular subtype (MST) on LRR and the benefits of PMRT are unclear. In this study, opposite results were found for patients with pN0 and pN1 tumors. Non-luminal type was a strong predictor of LRR for patients with one to three positive nodes, but was not associated with LRR risk in node negative patients. Truong et al. grouped 1994 patients with T1-2N0 disease into the same five molecular subtypes as this study: luminal A, luminal B, luminal HER2, HER2 enriched, and triple negative breast cancer [11]. Multivariate analysis revealed that no subtype was significantly associated with LRR. The 5-year LRR rate was 1.8% for luminal A, 3.1% for luminal B, 1.7% for luminal HER2, 1.9% for HER2 enriched, and 1.9% for triple negative tumors. Selz et al. constructed similar molecular subtypes for 508 pT1-3N0 patients, including luminal A, luminal B, HER2, triple negative, and unknown [13], and no subtype was found to be associated with increased LRR risk after multivariate analysis. In addition, Selz et al. found that Ki-67 expression >20% was an independent predictor of LRR within this cohort (Hazard Ratio, 4.18; *p* < 0.02). Among patients with one to three positive LNs treated with mastectomy alone, Moo et al. reported that molecular subtype was not an independent predictor of LRR (*p* = 0.38) [18]. However, Yang et al. showed that pT stage, two to three positive LNs, and molecular subtype were the independent risk factors for LRR in postmastectomy patients with pN1 [24].

The LRR rate is an important factor for selecting adjuvant RT. The use of PMRT is not recommended for patients at low risk for LRR <10% [25]. The St. Gallen recommendations indicate that patients with a 10-year LRR rate of 20% or more require PMRT [26], which means that quantification of LRR rate is important. Concerns about treatment-related morbidity and mortality, particularly in terms of cardiovascular disease and second malignancy, have discouraged the routine use of RT in all but high-risk groups. In our study, although the 10-year LRR rate in postmastectomy pN0 patients who did not undergo RT was 3%, subgroup analysis found that the risk of recurrence in high-risk patients was significantly higher than that of low-risk patients (10-year LRR rate, 13.3% vs. 0%; *p* < 0.001). In pN1 patients negative for HR, the 10-year LRR rate was 35.4% compared to 3.6% for those positive for HR. PMRT could benefit patients with pT1–2N0-1M0 breast cancer who are at high risk for LRR. The DBCG revealed a clear benefit from PMRT in terms of lowering LRR, especially in HR positive and triple negative subtypes [27]. The 10-year LRR rates were 15% in PMRT group compared to 32% in no PMRT group for triple negative breast cancer (p = 0.001). Patients received PMRT had better outcome than women without radiation in triple-negative stage I-II breast cancer after mastectomy in a prospective study [28]. Tendulkar et al. showed PMRT offers excellent control for patients with 1–3 positive LN, with no locoregional failures to date [29]. The 5-year rate of LRR was 8.9% without PMRT vs 0% with PMRT (p = 0.004). Yu et al. reported that postmastectomy supraclavicular RT may reduce the risk of LRR in patients with high-risk pN1 breast cancer [30]. Even with wide discrepancies in risk factors and LRR rates among previous studies and the present report, it is obvious that early-stage breast cancer patients treated with total mastectomy are not a homogeneous disease group. To address these variations and identify the early-stage patients that might benefit from PMRT, further large-scale studies are needed to investigate LRR risk factors in patients with pT1-2N0-1 breast cancer treated with mastectomy but not RT.

This study has several limitations, including its retrospective nature. The sample size of the pN1 cohort was small, which may have limited statistical power. The limited number of patients is why we used luminal vs. non-luminal subtypes for analysis instead of separate molecular subtype. Because there was insufficient evidence to rank the individual factors by their impact on LRR, we assessed the risk of LRR by the number of risk factors a patient had. In future studies, a predictive model, such as a nomogram, should be developed using a large number of patients. This would allow for the probability of LRR to be accurately calculated based on the numerical contribution of a number of risk factors.

In conclusion, our results demonstrate that not all patients with T1–2N0-1M0 breast cancer require PMRT. However, PMRT may be of value for node negative patients aged less than 50 years who do not receive adjuvant chemotherapy, and in patients with HER2-enriched or triple negative subtype tumors with one to three positive nodes.
